# Meaningful digital biomarkers derived from wearable sensors to predict daily fatigue in multiple sclerosis patients and healthy controls

**DOI:** 10.1016/j.isci.2024.108965

**Published:** 2024-01-18

**Authors:** Max Moebus, Shkurta Gashi, Marc Hilty, Pietro Oldrati, PHRT author consortium, Christian Holz

**Affiliations:** 1 Department of Computer Science, ETH Zürich, Stampfenbachstrasse 48, 8092 Zürich, Switzerland; 2 ETH AI Center, ETH Zürich, OAS J17, Binzmühlestrasse 13, 8092 Zürich, Switzerland; 3 Neuroimmunology Department, University Hospital Zürich, Frauenklinikstrasse 26, 8091 Zürich, Switzerland; 4 Competence Center for Rehabilitation Engineering and Science, ETH Zürich, Gloriastrasse 37/39, 8092 Zürich, Switzerland

**Keywords:** Health sciences, Neuroscience, Bioelectronics

## Abstract

Fatigue is the most common symptom among multiple sclerosis (MS) patients and severely affects the quality of life. We investigate how perceived fatigue can be predicted using biomarkers collected from an arm-worn wearable sensor for MS patients (n = 51) and a healthy control group (n = 23) at an unprecedented time resolution of more than five times per day. On average, during our two-week study, participants reported their level of fatigue 51 times totaling more than 3,700 data points. Using interpretable generalized additive models, we find that increased physical activity, heart rate, sympathetic activity, and parasympathetic activity while awake and asleep relate to perceived fatigue throughout the day—partly affected by dysfunction of the ANS. We believe our analysis opens up new research opportunities for fine-grained modeling of perceived fatigue based on passively collected physiological signals using wearables—for MS patients and healthy controls alike.

## Highlights

• Digital biomarkers predict fatigue ratings continuously throughout the day
• Physical and ANS activity while awake and asleep relate to fatigue ratings
• Dysfunction of the ANS alters the effects of biomarkers on fatigue ratings

## Graphical abstract

**Figure:**
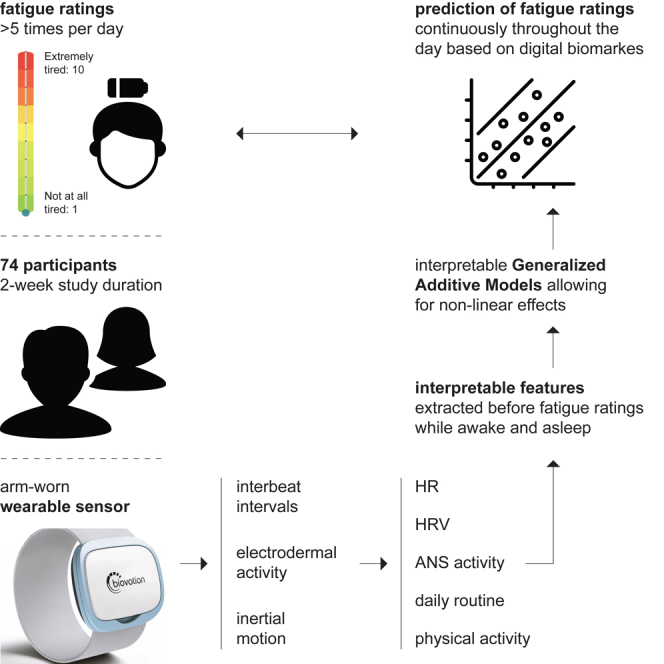

## Introduction

Fatigue is the most common symptom of multiple sclerosis (MS) patients. An estimated 53–87% of MS patients are severely impacted by fatigue.^1^ For MS patients, fatigue significantly impacts quality of life besides mood, sleep quality, and experienced functional independence.^2^^,^^3^^,^^4^^,^^5^ Even though fatigue has been a topic of research already in the 19^th^ century, no current definition suits all connected fields of research.^6^ As a symptom of “long covid”, currently still affecting 11% of all individuals ever infected with COVID-19 in the US, understanding fatigue and perceived fatigue has gained recent attention.^7^ In the context of MS, primary fatigue refers to fatigue caused by inflammatory activity (lesions), while secondary fatigue refers to fatigue related to symptoms of MS, such as low sleep quality or pain.^8^ Research has further distinguished between perceived fatigue (also known as state fatigue), a subjective assessment of fatigue potentially also influenced by general mood, and fatigability. Despite it being the most frequent symptom of MS, perceived fatigue is still poorly understood. Thus far, perceived fatigue has been the topic of numerous studies.^8^ However, respective studies either included only very few instances for MS patients or gaps of weeks or months in between consecutive assessments. Intensive longitudinal studies involving multiple assessments of fatigue per day across weeks or months are currently lacking.

Whereas fatigue in MS patients is commonly assessed using questionnaires, such as, e.g., the Fatigue Severity Scale (FSS)^9^ and the Fatigue Scale for Motor and Cognitive Functions (FSMC),^10^ fatigability is commonly assessed using mechanic devices such as a knee^11^ or hand-grip dynamometers.^12^ While the FSS, for instance, assesses fatigue over a prolonged period of time resulting in a measure of, so-called, trait fatigue,^13^ ratings on a visual analog scale (VAS) assess state fatigue, which forms a more subjective metric relating to a momentary perception of fatigue.^14^ State fatigue as captured via VAS ratings does not necessarily correlate strongly with fatigue captured by handgrip strength, for instance.^15^ VAS ratings capture also more subtle changes in state fatigue, for instance, due to excessive exercise, and thus change throughout the day.^16^^,^^17^ To fully understand its drivers, state fatigue thus has to be assessed continuously in the daily lives of MS patients.

Biosignals promise valuable insights when continuously assessing state fatigue. The activity of the autonomic nervous system (ANS), for instance, has previously been linked to state fatigue.^14^^,^^18^ For MS patients, the ANS is often impaired and symptoms of impairment might be observed already 10 years before the diagnosis.^19^^,^^20^ Symptoms of an impaired ANS have previously been linked to MS-related fatigue and how patients rank, for instance, on the Modified Fatigue Impact Scale.^21^ Given how the activity of the ANS varies due to emotion or stress,^22^ we hypothesize that changes in ANS activity also affect VAS fatigue ratings. Furthermore, given that the ANS is dysfunctional in a substantial proportion of MS patients, we hypothesize VAS fatigue ratings should be modeled separately for healthy controls, MS patients with a functional ANS, and MS patients with a dysfunctional ANS.

Wearable sensors allow to measure various biosignals including cardiac and electrodermal activity as well as skin temperature. Such devices have been extensively used to measure emotions and stress. Currently, state fatigue for MS patients has not been assessed using wearable sensors. Given the success of modeling other subjective responses, wearable sensors used in intensive longitudinal studies seem promising to model state fatigue. Besides improving our understanding of a very subjective phenomenon, this will contribute to individuals affected by fatigue increasing their quality of life.

In a two-week intensive longitudinal study, we investigate how also smaller changes in state fatigue can be explained by changes in behavior and biosignals for MS patients and a control group. For 74 participants, we model state fatigue multiple times a day totaling on average more than 50 observations per participant. We use an arm-worm wearable sensor collecting information about physical, cardiac, and electrodermal activity, as well as skin temperature and the weather.

To reveal the effect of changes in collected biosignals, we normalized biosignals and VAS fatigue ratings per participant and used interpretable generalized additive models (GAMs) recursively removing features without explanatory power. For MS patients, state fatigue levels were more difficult to model and changes in ANS activity seemed less informative toward state fatigue. Our study further revealed that while physical activity in terms of walking might reduce fatigue for healthy individuals, it might increase state fatigue for MS patients. Various metrics of cardiac activity while asleep were calculated to impact state fatigue the following day, highlighting the need to analyze the relationship between sleep and state fatigue more thoroughly for MS patients and healthy individuals alike.

## Results

We analyzed 3,733 VAS fatigue ratings from 74 individuals across a period of two weeks. We first analyzed how average VAS fatigue ratings and FSMC scores relate for MS patients in the VAS fatigue ratings and their relation to FSMC scores section. Then, after briefly comparing the overall distribution of VAS fatigue ratings in different subgroups in the VAS fatigue ratings among different subgroups section, we normalized VAS fatigue ratings and biosignals recorded from an arm-worn wearable sensor per participant as input to predictive models in the performance comparison of generalized additive models section. Using interpretable GAMs in the calculated effects on perceived fatigue section, we analyzed and interpreted predictors of state fatigue normalized per participant.

### Participants

We analyzed VAS fatigue ratings for healthy controls (Co, n = 23), MS patients with a functional ANS (MS I, n = 27), and MS patients with a dysfunctional ANS (MS II, n = 24). We classified the ANS of MS patients who scored higher than 17 on the abbreviated COMPASS questionnaire as dysfunctional.^23^^,^^24^ Table S3 compares heart rate variability metrics between MS patients with a dysfunctional ANS and MS patients with a functional ANS in more detail. Baseline characteristics for the three different subgroups are listed in Table 1.

**Table 1:** Comparison of healthy controls, MS patients with a functional ANS (MS I) and MS patients with a dysfunctional ANS (MS II)

	Co	MS I	MS II	$p_{C-I}$	$p_{C-II}$	$p_{I-II}$
N	23	27	24			
Female	13 (57%)	21 (78%)	13 (38%)	0.115	0.882	0.079
SPMS	0 (0%)	4 (15%)	4 (17%)			0.580
RRMS	0 (0%)	23 (85%)	20 (83%)			0.869
Age	33.9 (10.7)	34.3 (7.9)	39.1 (11.3)	0.487	0.095	0.125
VAS	3.8 (1.0)	3.6 (1.4)	4.5 (1.5)	0.271	0.083	0.026
FSMC	30.9 (9.0)	47.3 (20.3)	63.2 (18.7)	0.193	0.002	0.037
FSMC: cognitive	15.6 (3.4)	21.9 (9.5)	32.9 (10.2)	0.395	0.009	0.003
FSMC: motoric	15.2 (5.9)	25.4 (11.0)	30.3 (10.1)	0.089	0.013	0.199
Handgrip (kg)	27.4 (3.6)	21.6 (6.0)	26.6 (9.4)	0.004	0.480	0.053
9-hole PT (s)	17.2 (1.6)	20.1 (4.9)	24.8 (7.3)	0.071	0.001	0.001
EDSS		1.9 (1.5)	2.7 (1.4)			0.021
ARMSS		4.1 (2.4)	5.0 (1.8)			0.177
Disease Duration (y)	0 (0)	6.7 (6.8)	10.4 (8.8)			0.150

Percentages and standard deviations are provided in brackets in the first and second part of the table, respectively. The p values corresponding to distribution-free Wilcoxon signed rank tests testing for mean differences between Co and MS I, Co and MS II, and MS I and MS II are given in columns $p_{C-I}$, $p_{C-II}$, and $p_{I-II}$, respectively. SPMS and RRMS refer to the number of participants with Secondary Progressive MS and Relapse Remitting MS, respectively. Overall scores on the FSMC are presented, as well as the scores on the cognitive and motoric subparts. Only 8 participants of the control group completed the FSMC, the handgrip strength, and the 9-hole peg (9-hole PT) test. Participants of the control group were not scored on the EDSS or the ARMSS. Further information about MS patients can be found in Tables S1–S3. Figure S2 shows the distribution of VAS fatigue ratings.

#### VAS fatigue ratings and their relation to FSMC scores

**Figure 1:**
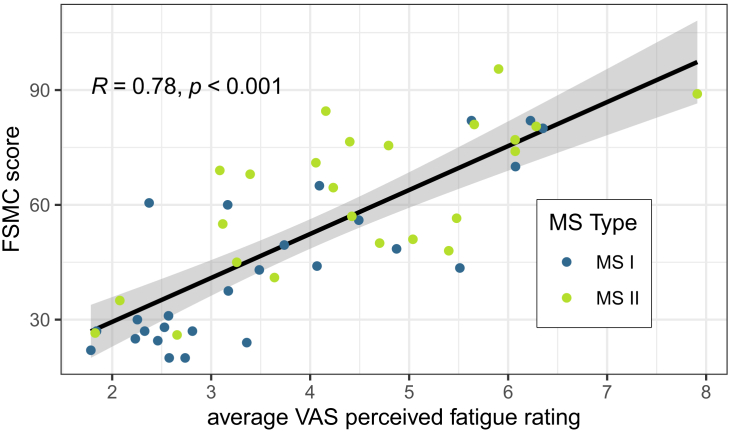
VAS fatigue ratings versus FSMC scores for MS patients MS I refers to MS patients with a functional ANS, while MS II refers to MS patients with a dysfunctional ANS. The Spearman correlation between VAS fatigue ratings and FSMC scores and its p value are given in the top-left corner. The fitted trendline with confidence interval is derived from a linear regression model. The overall distribution of VAS fatigue ratings is displayed in Figure S2.

For MS patients, we found that average VAS fatigue ratings correlate strongly with reported FSMC scores (Spearman correlation of 0.78). Figure 1 shows a clear trend between average VAS fatigue ratings and FSMC scores.

#### VAS fatigue ratings among different subgroups

Between the control group and MS patients, we found differences in the distribution of VAS fatigue ratings. Table 1 displays average baseline characteristics of the healthy controls (Co), MS patients with a functional ANS (MS I) and MS patients with a dysfunctional ANS (MS II), and whether the differences are statistically significant. Table 1 shows that VAS fatigue ratings significantly differ between MS patients with a dysfunctional ANS and MS patients with a functional ANS (p = 0.026). The two groups also significantly differ in terms of EDSS and FSMC scores, as well as heart rate variability (HRV) metrics (Table S3 in the Appendix). FSMC scores also significantly differ between MS patients with a dysfunctional ANS and the control group but not MS patients with a functional ANS and the control group.

#### Performance comparison of generalized additive models

With GAMs, we choose an interpretable class of models that have shown great flexibility for tabular data. Table 2 compares the performance of a GAM to other commonly used techniques such as a generalized linear model (GLM), (boosted) random forest regressor (RF and BRF), a support vector machine (SVM), and a small fully connected feedforward neural network (NN) for the prediction of VAS fatigue ratings. Removing all data points with missing values (No mean imp.), the GAM achieved the highest explained variance with 26.2% followed by a random forest with 26.1%. When imputing missing values by each participant’s mean feature value (Mean imp.), the SVM performed best with an explained variance of 24.6% followed by a GAM with 24.0%.

**Table 2:** Comparison of different modeling techniques for per-participant normalized VAS fatigue ratings

Model	No mean imp.			Mean imp.		
	$R_{\%}^{2}$	MAE	RMSE	$R_{\%}^{2}$	MAE	RMSE
RF	26.1	0.69	0.86	24.0	0.71	0.89
BTE	26.0	0.69	0.86	23.6	0.71	0.89
SVM	25.8	0.68	0.85	24.6	0.70	0.88
NN	24.4	0.71	0.88	23.1	0.72	0.90
GAM	26.2	0.69	0.85	24.2	0.70	0.88
GLM	25.9	0.68	0.84	24.0	0.70	0.88

For all participants, we compare the performance of a GAM to a random forest (RF),^25^ a boosted tree ensemble (BTE),^26^ a linear support vector machine (SVM),^27^ and a neural network (NN).^28^ We evaluate all techniques when removing all data points with missing values (No mean imp.) and imputing each missing value by the mean feature value per participant (Mean imp.). Details in the STAR Methods section.

Table 3 displays the performance of GAMs fitted as outlined in the STAR Methods section for five different subgroups of participants: all participants, the control group, all MS patients, and MS patients with and without a dysfunctional ANS. The model selected for the control group (within-group validation) performs best with an explained variance of 33.6% and a mean absolute error of 0.63, while the model selected for all MS patients at once performed worst. When performing across-group evaluation, model performance drops in all cases. Model performance ($R^{2}$) drops by almost 16% when evaluating the model optimized for the control group on MS patients with a functional ANS (MS I) (from 33.6% to 17.7%). The difference in $R^{2}$ is statistically significant (p<0.001). Similarly, the performance of models trained on data of MS patients with a functional ANS drops significantly when used to predict VAS fatigue ratings of MS patients with a dysfunctional ANS, and vice-versa.

**Table 3:** Performance evaluation of GAMs fitted for five different groups

Group		P	N	Imp.	No mean imp.			Mean imp.			Baseline			*p*	
Train	Test				$R_{\%}^{2}$	MAE	RMSE	$R_{\%}^{2}$	MAE	RMSE	$R_{\%}^{2}$	MAE	RMSE	*p*	$p_{imp}$
All	All	74	3733	25%	26.2	0.69	0.85	24.2	0.70	0.88	0	0.78	0.99		
Co	Co	23	1137	20%	33.6	0.63	0.78	29.8	0.66	0.84	0	0.80	0.99		
	MS I				17.7	0.73	0.91	18.1	0.73	0.91	0	0.83	0.99	<0.001	<0.001
	MS II				18.2	0.74	0.92	17.1	0.75	0.93	0	0.81	0.99	<0.001	<0.001
MS	MS	51	2596	27%	25.9	0.68	0.86	22.2	0.71	0.89	0	0.83	0.99		
MS I	MS I	27	1338	27%	27.5	0.67	0.84	24.8	0.70	0.88	0	0.80	0.99		
	Co				18.6	0.74	0.89	18.2	0.73	0.88	0	0.80	0.99	<0.001	<0.01
	MS II				17.5	0.73	0.93	14.2	0.75	0.94	0	0.81	0.99	<0.001	<0.001
MS II	MS II	24	1258	28%	27.8	0.69	0.85	22.3	0.71	0.90	0	0.81	0.99		
	Co				25.9	0.67	0.83	21.2	0.70	0.86	0	0.80	0.99	0.05	0.36
	MS I				20.6	0.72	0.89	17.2	0.74	0.93	0	0.80	0.99	<0.001	<0.01

P: number of participants, N: number of observations, Imp.: % of observations with mean imputation. Models are trained based on data of participants in the Train group, and evaluated on participants in the Test group. Each participant’s average VAS fatigue rating is used as a baseline regressor for comparison. The metrics are averaged across all participants of the Test group. In case the model is tested on participants of the same group it is trained on (within-group evaluation), each participant is left out from the training set once for testing (leave-one-participant-out cross-validation). In case the model is tested on participants of a different group than it is trained on (across-group evaluation), the model is trained on data from all participants in the Train group and evaluated on data from each participant of the Test group separately to match the setting of the within-group evaluation. The *p* and $p_{imp}$ columns give the p value of Wilcoxon signed rank tests indicating whether the across-group performance is different from the within-group performance. At each split, the model is trained omitting any data points with imputed data and evaluated once on this reduced dataset without any mean imputation (No mean imp. & *p*) and once including mean imputation (Mean imp. & $p_{imp}$).

Table 3 shows varying proportions of incomplete data points from 20 to 28% between subgroups, where missing data points were mean imputed (Mean imp.). To prevent inaccurately detected heartbeats from our optimal sensor worn at the arm, we require 5-min windows with little motion when participants were at rest to calculate metrics about ANS activity. Missing 5-min low-motion windows in a 1-h window prior to a VAS fatigue rating is the most frequent reason for incomplete data points—see the STAR Methods section for more details.

#### Calculated effects on perceived fatigue

Table 4 lists all significant effects for the VAS fatigue ratings and their directions for the three different groups: the control group (Co), MS patients with a functional ANS (MS I) according to COMPASS,^23^^,^^24^ and MS patients with a dysfunctional ANS (MS II). Table 4 summarizes the effects calculated for the aforementioned groups. The effects are derived from partial independence plots (see Figure S1 as an example).

**Table 4:** Summary of the effects of an increase in the respective variable on the VAS fatigue rating

Variable			Effect on VAS rating		
			Co	MS I	MS II
time of day			╱		╱
time spent awake				╱	
sleep duration				╲	
HR	6h:	mean	╱		
	6h:	sd		╱	
	as:	mean			╱
	as:	sd	╲		
	as:	slope	╲	╱	
SD1	3h:	min			╲
	3h:	mean			╱
	aw:	mean	╲		
	aw:	slope	╲		╱
	as:	mean	╲		
	as:	sd	╱		
SD2	3h:	min	╲		
	aw:	sd		╱	
	aw:	slope			╲
	as:	min	╱		
	as:	sd			╱
SDNN	1h:	sd			╱
	3h:	slope	╱		
	6h:	mean	╱		
	aw:	max		╲	
	as:	min		⌢	
	as:	max		╱	
	as:	sd		╲	
EDA peaks	1h:	sd			╱
	6h:	sd	╲		
steps	6h:	count	╲	╲	
	aw:	count			╱
activity	6h:	sd			╱
	6h:	max		╲	
	aw:	max	╲	∼	╲
	aw:	sd		╱	
	aw:	int.	╱		╱
	as:	max		╱	
temp	day:	mean		╲	
	day:	min			╲
temp (felt)	day:	min		╱	
dew	day:	mean	╲	╲	
humidity	day:	mean			╱

The symbols ╱, ╲, ⌢, ⌣ and ∼ summarize the shape of respective partial dependence plots. ‘1h’, ‘3h’, ‘6h’, ‘aw’, and ‘as’ refer to the 5 time horizons defined in the STAR Methods section of one, three, and 6 h prior to the respective rating, and awake and asleep, respectively. Variables connected to weather were calculated across the whole day. Co = control group; MS I = MS patients with a functional ANS; MS II = MS patients with a dysfunctional ANS.

Predictors for state fatigue include variables related to participants’ daily routine, cardiac activity, electrodermal activity, physical activity, and the weather. While most variables were calculated over longer time horizons such as the time period when participants were asleep or since they woke up before a VAS fatigue rating 6 predictors are variables collected within 3 h before a VAS fatigue rating.

#### Ablation study for perceived fatigue

We conducted an ablation study to better understand how different groups of features contribute to model performance for the five subgroups of the dataset. We find that removing feature groups when modeling VAS fatigue ratings for all participants at once (All), or all MS patients at once, does not significantly change performance. For MS patients with a dysfunctional ANS (MS II), the explained variance decreases when removing any combination of features (by up to 6%). When removing all four feature groups, $R^{2}$ decreases the most for MS patients with a dysfunctional ANS (4.9%).

## Discussion

### Fatigue and state fatigue

According to a commonly used definition by Kluger et al.,^29^ there are two distinct types of fatigue. The first type, known as performance fatiguability, describes general weakness of muscles or the inability to perform a task at the same speed or quality for long. It is an objective usually stationary assessment of (trait) fatigue. It is commonly assessed via questionnaires such as the FSMC,^10^ or mechanic devices such as a knee^11^ or hand-grip dynamometer.^12^ Recently, however, also mobile devices have been used to assess fatiguability.^30^ Perceptions of fatigue (state fatigue), as assessed via VAS ratings, form the second type of fatigue as defined by Kluger et al., which does not necessarily relate to the first type. While these two types of fatigue have to be treated separately and do not have to occur at the same time, they can influence each other and might be observed together. With a correlation of 0.79 (Figure 1), we find that MS-related trait fatigue, as assessed via the FSMC, relates well to state fatigue as assessed via the average VAS fatigue rating recorded during our two-week study. This does not imply, however, that the smaller, short-term changes in fatigue we model after normalizing VAS fatigue ratings per participant also relate to changes in trait fatigue. These more subtle changes are intertwined with a feeling of tiredness as often included in the definition of perceived fatigue.^31^ This is also supported by our finding of a strong positive effect of time of day on state fatigue and time spent awake on state fatigue.

Table 1 further shows that FSMC scores, as well as VAS fatigue ratings, differ significantly between the control group and MS patients with a dysfunctional ANS, as well as between MS patients with a functional ANS and MS patients with a dysfunctional ANS, highlighting fatigue severity for MS patients with a dysfunctional ANS. While the average FMSC score of MS patients with a functional ANS (47.3) lies above the cut-off value of 43 for mild fatigue,^10^ the average FSMC score of the control group (30.9) is not statistically different. Consequently, while there is evidence of (trait) fatigue in MS patients with a functional ANS given the average FSMC scores of 47.3, the difference to the healthy controls is much less substantial than for MS patients with a dysfunctional ANS. Similarly, there is also no significant difference in VAS fatigue ratings between the control group and MS patients with a functional ANS. Thus, between healthy controls and MS patients with a functional ANS, there is no general difference in perceived fatigue (state fatigue), and VAS fatigue ratings might primarily relate to a feeling of tiredness for MS patients with a functional ANS.

### Performance by different modeling techniques

For all models, we observed lower performance when mean-imputing missing values. This is expected since we add noise by doing so. The GAM achieved the highest explained variance when all observations with mean imputed variables were removed and generally performed on par with all other modeling approaches. Differences in $R^{2}$, MAE, and RMSE between different approaches, however, are overall small. GAMs allow for the interpretation of calculated effects, while little to no predictive performance on completely unseen test sets seems lost in comparison to less interpretable techniques such as the SVM, RF, BTE, or NN.

### Performance in different sub-groups

Modeling the control group on its own resulted in the best performance as displayed in Table 3. For MS patients, this highlights a lower signal-to-noise ratio given the observed variables. The significant drops in performance in Table 4 when a model fitted for MS patients with a functional ANS is used to predict VAS fatigue ratings for MS patients with a dysfunctional ANS, and vice-versa, highlight that state fatigue seems to respond differently to changes in biosignals based on ANS dysfunction. This matches observed differences in the sets of predictors in Table 4.

### Ablation study

We evaluated the GAM for modeling VAS fatigue ratings when removing combinations of feature groups for the five different subgroups of participants as part of an ablation study. Table 5 displays the performance of the GAM fitted on combinations of the four feature groups: including all available features, removing one group, removing all groups apart from one, and removing all groups. We further tested whether adding any combination of features changes model performance significantly compared to removing all four feature groups in the column on the very right.

**Table 5:** Ablation study for VAS fatigue ratings

Features	CAR EDA ACC WEA	EDA ACC WEA	CAR ACC WEA	CAR EDA WEA	CAR EDA ACC	CAR	EDA	ACC	WEA	

ALL	26.2	26.2	26.1^∗∗^	26.5	26.5	26.3	26.4	26.8	26.0	26.6
Control	33.6^∗∗∗^	29.7	33.1^∗∗∗^	32.3^∗^	33.7^∗∗∗^	30.1	31.3^∗^	29.4	29.4	29.8
MS	25.9	25.3^∗^	25.1	24.5	25.8	24.5	24.0	25.2	24.8	24.6
MS I	27.6^∗∗^	26.2^∗^	27.6^∗∗∗^	24.0^∗∗^	27.8^∗∗∗^	25.3	24.8	25.6	26.5^∗∗^	25.6
MS II	27.8^∗∗∗^	23.3	25.9^∗∗∗^	22.8	26.9^∗∗∗^	21.8	23.1	22.7	22.7	22.9

We assessed model performance in terms of explained variance ($R^{2}$) when removing one of the four feature groups: cardiac activity (CAR), electrodermal activity (EDA), physical activity (ACC), or weather (WEA). We further assessed model performance when removing all of the four feature groups, and all apart from one. Features related to participants’ daily routine (time of day, sleep duration, etc.) were always included and they form the baseline performance in the very right column. We tested whether model performance differs significantly from this baseline in the very right column using Wilcoxon signed rank tests across 100 perturbations of participants. Differences in performance at a significance level of p<0.05, p<0.01, and p<0.001 are marked with ^∗^, ^∗∗^, and ^∗∗∗^.

When removing all four feature groups, VAS fatigue ratings are modeled only based on features related to daily routines such as the time of day or sleep duration, which resulted in the highest performance when modeling all participants at once. The high performance when removing all feature groups indicates that the calculated effects might not generalize well across all subgroups of participants. For MS patients with a dysfunctional ANS, we observed the greatest improvement in explained variance when including all four feature groups (27.8 versus 22.9). This highlights that for MS patients with a dysfunctional ANS changes in biosignals might have the strongest effect among the three subgroups. Adding the four feature groups resulted in significantly higher model performance only when modeling the control group, MS patients with a functional ANS and MS patients with a dysfunctional ANS separately. This highlights that while changes in biomarkers seem to affect VAS fatigue ratings for all participants, the effects likely do not generalize across the three groups. Generally, adding all four feature groups affects $R^{2}$ by at most 4.9% (MS II), highlighting that features about participants’ daily routines are already very informative toward VAS fatigue ratings.

### Daily routine

For all three subgroups of participants, either the time of day or the time that passed since participants last woke up were selected as predictors of VAS fatigue ratings indicating that VAS fatigue ratings increase the longer participants are awake. Only for MS patients with a functional ANS, sleep duration was selected as a predictor for state fatigue indicating a potentially increased importance of sleep toward state fatigue.

### Cardiac activity

We find various significant effects related to cardiac and ANS activity. However, they are not always consistent across all subgroups as outlined in the following sections in more detail.

#### Heart rate

For MS patients with a dysfunctional ANS, only the average heart rate (HR) while asleep was calculated to increase state fatigue, matching previous studies for patients with chronic fatigue syndrome.^32^ Interestingly, a more positive trend of HR while asleep was calculated to reduce state fatigue for the control group but increase state fatigue for MS patients with a functional ANS.

#### Heart rate variability: Overall activity

We have found various effects related to HRV metrics for the three groups of participants. There are many potential reasons, as to why ANS activity and, subsequently, HRV metrics might change. This includes momentary effects of deep breathing,^33^ as well as disease progression of MS patients in the long run.^19^ HRV metrics also vary between different sleep stages and due to stress, pain, or changes in mood.^34^ Given that we aggregate HRV metrics across the course of 1 h to a maximum of roughly 16 h, stress, changes in mood, pain, physical activity, or sleep stages are likely causes for changes in HRV metrics.

SDNN is a measure of overall HRV influenced by both the sympathetic and parasympathetic part of the ANS. For the control group, we find that SDNN metrics up to 6 h prior to a VAS fatigue rating affect state fatigue. For MS patients with a dysfunctional ANS, the calculated effect of increased variability in SDNN activity during 1 h prior to a VAS fatigue rating hints at an immediate effect of changes in ANS activity that increase state fatigue. For MS patients with a functional ANS, increases in maximum SDNN while awake are calculated to decrease state fatigue, while increases in maximum SDNN while asleep are calculated to increase state fatigue the following day.

#### Heart rate variability: Parasympathetic activity

Besides measuring general ANS activity, SD1 provides information about the activity of the parasympathetic part of the ANS, responsible for relaxation after stress (rest and digest).

Interestingly, parasympathetic activity does not seem to affect state fatigue for MS patients with a functional ANS. For MS patients with a dysfunctional ANS, however, average and minimal parasympathetic activity impact state fatigue up to 3 h before a VAS fatigue rating. Further, the constructed GAMs indicate that increased parasympathetic activity increases state fatigue for MS patients with a dysfunctional ANS but decreases it for the control group. For the control group, a decrease in state fatigue given increased parasympathetic activity might be linked to the reaction of the ANS to mood changes. Parasympathetic activity increases given a positive mood, which seems to contradict for MS patients with a dysfunctional ANS. While asleep, parasympathetic activity was calculated to affect state fatigue throughout the following days only for the control group, hinting at a possible relationship to sleep behavior and the time spent in different sleep stages.^35^

#### Heart rate variability: Sympathetic activity

SD2 is a measure of general ANS activity but more specifically is influenced by activity of the sympathetic part of the ANS—responsible for alertness in dangerous or stressful situations (fight or flight response). For the control group, increases in minimal SD2 while asleep and awake (3h prior) are linked to increased VAS fatigue ratings indicating a relationship between experienced stress and state fatigue. While increases in variability of sympathetic activity while awake are calculated to increase state fatigue for MS patients with a functional ANS, a positive trend in sympathetic activity is linked with decreased state fatigue for MS patients with a dysfunctional ANS. For increased variability while asleep, however, we find that it is linked to increased VAS fatigue ratings throughout the next day for MS patients with a dysfunctional ANS. Given the variability of HRV metrics while asleep, we believe the sleep behavior of MS patients and their reaction to negative emotions such as stress should be studied more closely in the future.

### Electrodermal activity

The variation in the number of peaks in the EDA signal was calculated to significantly affect VAS fatigue ratings for MS patients with a dysfunctional ANS and the control group. For MS patients with a dysfunctional ANS, a higher variability in the number of peaks during 1 h prior to the VAS fatigue rating was calculated to increase state fatigue. For the control group, higher variability in the number of EDA peaks while asleep was calculated to decrease state fatigue the following day. EDA peaks, especially if they form, so-called, EDA storms indicate arousal. While asleep, they are most common in non-REM sleep phases,^36^ potentially highlighting the importance of different sleep phases and completed cycles for lower state fatigue for healthy individuals. While awake, EDA peaks indicate cognitive and emotional stress,^34^ indicating that MS patients with a dysfunctional ANS might be particularly affected by stressful events. Further, EDA is an alternative biomarker for sympathetic activity. For MS patients with a dysfunctional ANS, the effects of EDA and SDNN are thus aligned.

### Physical activity

For MS patients and the control group, we generally observed that an increase in physical activity (step count or total movement) while awake increases state fatigue. An increase in maximum total acceleration of the arm is associated with decreased fatigue for all participants, however. Since arm movement might be observed also while the participants sit, this might indicate that not all types of physical activity increase state fatigue and that walking might, for instance, decrease it.

While asleep, variables about physical activity might relate to sleep continuity. For MS patients with a functional ANS, the associated increase in state fatigue throughout the day due to an increase in the maximum acceleration of the arm observed while asleep might be related to decreased sleep continuity.

### Weather

The weather, and temperature in particular, was shown to impact MS patients’ cognitive and motor skills.^37^ It is thus not surprising that minimal and mean temperature was selected as a predictor for VAS fatigue ratings for MS patients with a dysfunctional ANS and MS patients with a functional ANS respectively. Interestingly, however, an increase in felt minimal temperature was associated with an increase in state fatigue for MS patients with a functional ANS hinting at a possible interaction between objective and felt minimal temperature.

For MS patients with a functional ANS and the control group, we found that days with increased amounts of dew were associated with decreased fatigue, while days with high humidity were associated with higher state fatigue for MS patients with a dysfunctional ANS.

### Concluding remarks

In this paper, we have highlighted that state fatigue can be modeled at a time resolution of multiple times a day for healthy individuals and MS patients alike. Based on passively collected data alone, our models clearly outperformed baseline regressors predicting each participant’s average response over our two-week study duration. Dysfunction of the ANS affects the relationship between biomarkers and state fatigue. For healthy individuals, MS patients with a functional ANS, and MS patients with a dysfunctional ANS, state fatigue thus has to be analyzed separately.

VAS fatigue ratings follow a daily upward trend and the time of day and the time participants spent awake were the strongest predictors for state fatigue. Deviations from this daily upward trend might be explained by changes in biomarkers related to cardiac, ANS, electrodermal, and physical activity. The calculated effects linked to the activity of the sympathetic nervous system indicate that emotional states, such as stressful or particularly calming experiences, might affect state fatigue. Further, we find changes in biosignals while asleep to predict state fatigue throughout the following day. This highlights that sleep behavior and its relation to state fatigue should be studied more closely for healthy individuals and MS patients alike.

### Limitations of the study

Toward the goal of analyzing fatigue in MS patients continuously, our study has several limitations. Firstly, all our findings need to be verified in more large-scale efforts representative of a broader population of MS patients. While the study is somewhat balanced between healthy controls, MS patients with a functional ANS and MS patients with a dysfunctional ANS, age and gender distributions are not fully matched within these groups and are unlikely to accurately represent the broader population. Secondly, while VAS fatigue ratings form a low-effort assessment of fatigue that is easy to integrate on mobile devices and does not pose a major effort for study participants, they do not necessarily link to other commonly used measures of fatigue such as FSMC,^10^ or EDSS.^38^ Besides fatigue, they might capture feelings of exhaustion due to recent physical exercise, for instance. Further, VAS fatigue ratings do not separate motoric and cognitive aspects of fatigue. For MS patients, VAS fatigue ratings capture what is often described as perceived fatigue or state fatigue. Thirdly, our study’s time horizon of 2 weeks is arguably too short to capture slower long-term changes in fatigue. We believe the continuous assessment of fatigue across long periods accompanied by wearable sensors would be a most interesting avenue for future research. Lastly, since our modeling approach using GAMs simply aims to identify biomarkers with large explanatory power toward VAS fatigue ratings, the causality of the effects derived in this fully observational study requires further assessment.

## STAR★Methods

### Key resources table

**Table:** 

REAGENT or RESOURCE	SOURCE	IDENTIFIER
**Software and Algorithms**		

R statistical software	The R foundation	Version 4.2.2
Mixed GAM Computation Vehicle with Automatic Smoothness Estimation	https://cran.r-project.org/web/packages/mgcv/index.html	Version 1.9.0

### Resource availability

#### Lead contact

Further information and requests for resources and reagents should be directed to and will be fulfilled by the lead contact, Christian Holz (christian.holz@inf.ethz.ch).

#### Materials availability

This study did not generate new unique reagents.

#### Data and code availability

• The data reported in this study cannot be deposited in a public repository due to the approved study protocal. The data reported in this study is available from the lead author upon reasonable request and signing a data-sharing agreement.
• This paper does not report original code.
• Any additional information required to reanalyze the data reported in this paper is available from the lead contact upon request.

### Experimental model and study participant details

We recruited 74 participants aged between 18 and 65 without concomitant diseases via convenience sampling at the neuroimmunology department outpatient clinic of the University Hospital Zurich, Switzerland. The study protocol was reviewed and approved by the Cantonal Ethics Committee of Zurich (SNCTP000003494). MS patients (n=51) and a control group (n=23) were recruited between the 29^*th*^ of November 2019 and the 29^*th*^ of July 2021. Upon inclusion, MS patients were scored on the Fatigue Scale for Motor and Cognitive Functions (FSMC), completed the COMPASS-31 questionnaire,^23^ measured their handgrip strength 10 times using a handgrip dynamometer (with the dominant hand), performed 4 consecutive 9-hole peg tests (2 with each hand), and reported their medical history including any medication they were taking. Also, 8 members of the control group performed 10 consecutive handgrip strength tests, 4 9-hole peg tests, and were scored on the FSMC. Overall, participants were 34.7 years old (SD=10.1), and 48 were female (sex assigned at birth). 32% of all participants scored higher than 17 on the abbreviated COMPASS questionnaire indicating dysfunction of the ANS.^23^^,^^24^ Baseline characteristics of all participants and information about MS patients’ medical condition can be found in Table 1. Table S3 further compares ANS activity between MS patients with a dysfunctional ANS and MS patients with a functional ANS while awake and asleep. Some MS patients were on disease-modifying treatment (DMT) or medication that is known to affect HRV metrics. We have listed the number of MS patients and the respective medication in the Appendix in Tables S1 and S2. Participants’ ancestry, race, ethnicity, education, and socioeconomic status were not reported.

### Method details

For the duration of two weeks, participants wore a wearable sensor (Everion, Biofourmis AG) on the arm recording HR at 1Hz, inter-beat-intervals (IBIs), skin temperature at 1 Hz, electrodermal activity at 1 Hz, step count, and total acceleration at 1 Hz. The Everion sensor in use demonstrated high agreement with a gold-standard Holter ECG device.^39^ We further collected information about the weather such as temperature (actual and felt), solar energy, cloud coverage, and time between sunrise and sunset. Participants were equipped with two wearable sensors and instructed to swap them for charging once per day. The respective sensor in use streamed data via Bluetooth to a smartphone upon which a custom-built application was installed. If necessary, participants were equipped with a Google Pixel 3a phone for the study duration.

Continually throughout the day, participants rated their level of fatigue on a VAS from one to ten. Participants were instructed to complete at least three VAS fatigue ratings daily. On average, every participant reported their perceived fatigue nearly 51 across the two-week study duration. The average rating was 3.9. Participants’ average ratings varied with a standard deviation of 1.4. On average, the VAS ratings varied with a standard deviation of 1.5 per participant. Figure S2 shows a histogram of all VAS fatigue ratings.

### Quantification and statistical analysis

#### Feature extraction

We extracted and aggregated features across five different time intervals before each VAS fatigue rating: during one hour prior, three hours prior, and six hours prior, while participants were asleep at night, and since participants woke up. Four out of the five intervals are identical, if a participant rated their fatigue level less than one hour after waking up. If a participant rated their fatigue level between one and three hours after waking up, three out of five intervals are identical. Given that participants reported on average 3.8 VAS ratings per day, features extracted for different VAS fatigue ratings will also be aggregated over partially overlapping time intervals.

Based on the inter-beat intervals (IBIs) from the arm-worn wearable sensor, we calculated heart rate variability (HRV) features according to recommendations of the HRV Task Force^40^ using the opne-source library ‘pyHRV’.^41^ We only computed HRV metrics during 5-minute windows with no measurable continuous arm motion, with less than 4 IBIs that we linearly interpolated due to artifacts,^42^ and when participants were at rest. We classified participants to be at rest when their heart rate was less than 55% of their maximum heart rate (220 BPM - age). To measure HRV, we calculated the standard deviation of the IBIs (SDNN), the standard deviation of the distance to the 45^°^ and -45^°^ line of the point-caré plot of consecutive IBIs (SD1 and SD2, respectively). We did not calculate RMSSD (root mean squared difference of consecutive IBIs), since it is mathematically identical to SD1.^43^ For HR and HRV features, we calculated the minimum, maximum, and average values as well as the standard deviation across the five time horizons outlined above. Additionally, we calculated the slope of the linear regression line of each variable fitted across these time intervals. We will refer to these regression coefficients as the slope of the respective signal over a given time interval.

Similarly, for the skin temperature and EDA signals, we extracted the slopes, standard deviation, as well as average, minimum, and maximum values across the five different time horizons outlined above. For the EDA signal, we also extracted the number of peaks.^44^

We summed the step count over all five time horizons as a measure of physical activity. We further extracted the average, minimal, and maximum values of the total magnitude of the accelerometer and also approximated the integral of the total magnitude over the five time horizons.

We estimated the sleep and awake times based on the accelerometer signal and the participants’ heart rates. In addition to defining the five time horizons based on the sleep and awake times, we also extracted further sleep-related features. In particular, we extracted the duration of participants’ sleep in the night before each VAS fatigue rating, the time that participants spent awake before each VAS fatigue rating, and if participants woke up or went to bed later or earlier than on average across the two weeks of the study.

#### Missing data imputation

At least one variable was missing in 927 out of all 3733 data points. This occurred most often when signals were aggregated over a single hour. Metrics about cardiac activity were missing particularly frequently since we required 5-minute windows with little motion when participants were at rest in order to accurately compute metrics about ANS activity. Synchronization errors between the two sensors that participants wore in alternating order are another reason for missing data. Per participant, we imputed missing values using their corresponding mean recorded value for each variable over the two weeks. We trained models on the dataset without any imputed values (2234 data points) and evaluated them on this smaller dataset as well as on the full dataset including data points with mean imputed variables. Both approaches (mean imputing and removing data points with missing information) are known to produce biased estimates if data are not missing completely at random. The suitability of either approach depends heavily on whether there is an underlying cause for missing data (i.e., non-random selection) that might influence the model fit.^45^ Recent literature has proposed several more sophisticated techniques for imputing missing information.^46^^,^^47^ In literature across various domains mean imputation is commonly used as a relatively simple imputation technique that has shown comparative performance to other imputation techniques.^48^^,^^49^^,^^50^ By evaluating our models without any imputation and with mean imputation, we aim to demonstrate the general feasibility of our approach. Assessing different imputation approaches to maximize the performance of our models might be an interesting route for future work.

#### Per-participant normalization

To analyze how relative changes in calculated features affect the VAS fatigue rating relatively compared to participants’ average ratings, we normalized the calculated features and the fatigue rating per participant. We did so by subtracting the mean value and dividing by the respective standard deviation per participant. We performed the normalization ignoring any missing values and imputed the missing values with zero, which equals the mean value per participant since we normalized per participant.

#### Generalized additive models

We modeled the data using Generalized Additive Models (GAMs).^51^ As so-called glass-box models with high flexibility, GAMs have been a popular modeling technique in the environmental sciences, health sciences, and recently also to model subjective responses based on passively collected sensor data.^52^^,^^53^^,^^54^ For our use case, we compare the performance of GAMs to Generalized Linear Models (GLMs), and commonly chosen modeling techniques such as random forests, support vector machines, and small neural networks.

We constructed the GAMs for the VAS fatigue rating using the “MGCV” library.^55^ We fitted all effects using smoothing splines, allowing for non-linear relationships. The non-linear effects were fitted as thin-plate regression splines, as generally recommended.^55^^,^^56^ We included a further penalty on the null space of the regression splines shrinking effects to zero and effectively removing them from the model in case of low explanatory power. This automatically performs variable selection and reduces the danger of overfitting—promoting a more robust fit.

We performed a sequential backward elimination procedure to extract the set of variables with explanatory power towards the VAS fatigue rating. At each iteration, we fitted the model on 1000 sub-samples of the data randomly removing all samples from two participants. Averaging the estimated degrees of freedom of the fitted smoothing splines and respective p-values across the 1000 splits, we removed all variables where the fitted smoothing spline required on average less than 0.1 estimated degrees of freedom. This removes all variables where the effect was automatically shrunk to zero on a large proportion of the splits due to low explanatory power. Further, if at least one variable had an average p-value higher than 0.05, we removed the variable with the highest average p-value across the 100 splits. We repeated the process until all included fitted effects required on average more than 0.1 estimated degrees of freedom and were significant at a significance level of 0.05. We then fitted the model on all participants at once, again now removing any effects according to the rules outlined above.

To compare the effects in different groups of the dataset and identify differences between MS patients, the control group, or different types of MS, we performed the model fitting process for five different groups. Once, we performed the model fitting process for all participants together (n=74), once for the control group (n=23), once for all MS patients (n=51), and once for MS patients with and without a dysfunctional autonomic nervous system, separately (n=24 and n=27, respectively).

#### Alternative learning algorithms

For the comparison of different modeling techniques in Table 2, we trained a random forest regressor (RF) sing the ‘Ranger‘ software package,^25^ a boosted tree ensemble regressor (BTE) using the ‘Model-Based Boosting‘ software pacakge,^26^ a linear support vector machine (SVM) using the ‘Kernel-Based Machine Learning Lab‘ software package,^27^ and a neural network (NN) suing Keras.^28^ For the RF, BTE, and linear SVM, we used the default settings as recommended by each software package. We optimized the neural network structure across two hidden layers with either 16, 32, or 64 neurons each. We found the highest performing using the smallest architecture of two hidden layers with 16 neurons each. For each alternative learning algorithm, we used the variables pre-selected in the iterative GAM fitting process described in the previous subsection of STAR Methods section. All model performances were averaged across 100 fitting processes where each time all observations of 1 randomly selected participant formed the test set.

## Supplemental information

Document S1. Figures S1 and S2 and Tables S1‒S3

Document S2. PHRT author consortium
