# Supplementary material for: Meaningful digital biomarkers derived from wearable sensors to predict daily fatigue in multiple sclerosis patients and healthy controls
Source: iScience. 2024 Jan 18;27(2):108965. doi: 10.1016/j.isci.2024.108965 (PMC10867654; doi:10.1016/j.isci.2024.108965)
Supplement: Document S1. Figures S1 and S2 and Tables S1‒S3 [file mmc1.pdf]

iScience, Volume *27*

Supplemental information

Meaningful digital biomarkers derived from wearable sensors to predict daily fatigue in multiple sclerosis patients and healthy controls

Max Moebus, Shkurta Gashi, Marc Hilty, Pietro Oldrati, PHRT author consortium, and Christian Holz

medication	MS I	MS II
None	4	4
Dimethylfumarat	3	2
Natalizumab	7	4
Ocrelizumab	7	4
Ozanimod	1	1
Rituximab	0	3
Siponimod	1	0
Teriflunomid	0	1
aHSCT	4	5

Table S1: **Numbers of participants who were on disease-modifying therapy (DMT) during the study per MS patient group, related to Table 1.**

medication	MS I	MS II
None	21	13
Betmiga	0	3
Citalopram	1	0
Cymbalta	0	3
Duloxetin	0	1
MetoZerok	0	1
Minoxidil	2	0
Ozanimod	1	1
Ritalin	0	1
Siponimod	1	0
Tamsulosin	0	1
Trittico	1	0

Table S2: **Numbers of participants who took medication known to affect HRV metrics during the study, related to Table 1.**

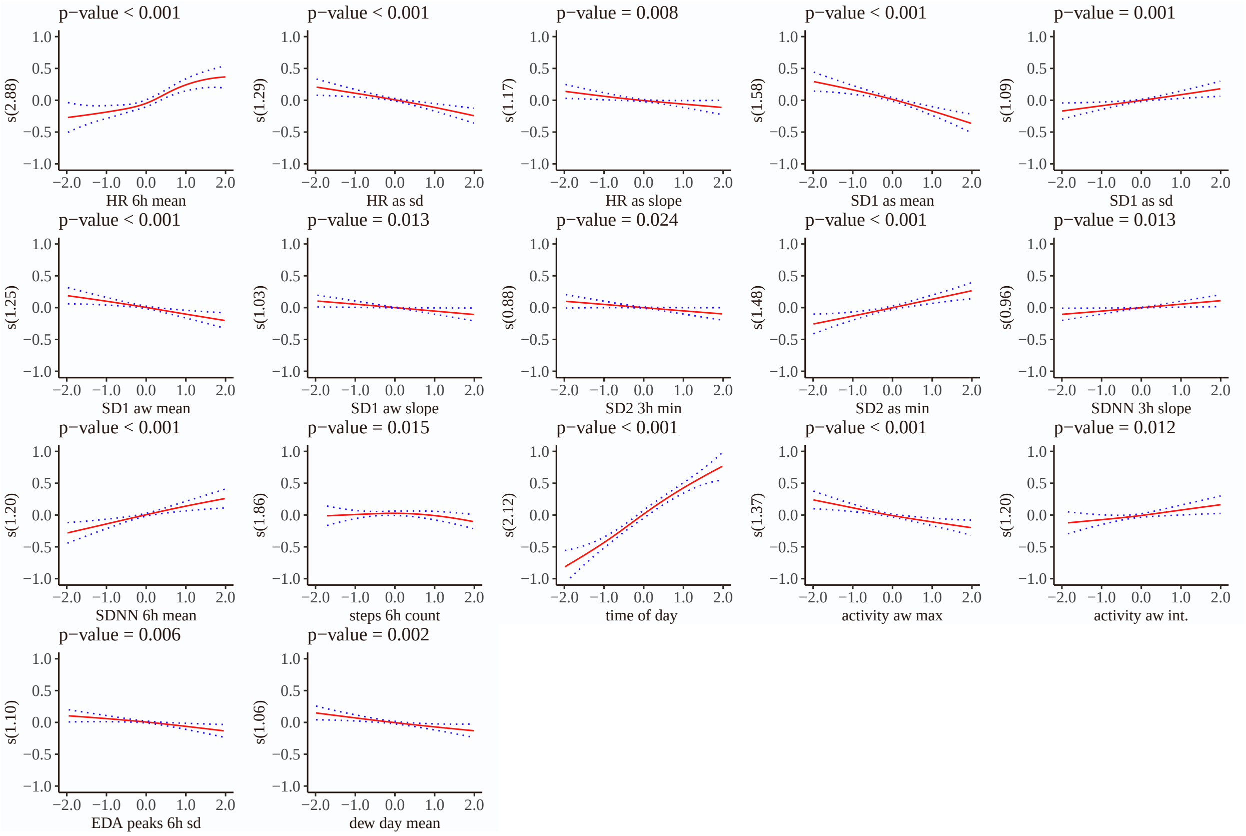

Figure S1: **Calculated effects on VAS fatigue ratings for the control group, related to Table 4.**
As an example, this Figure displays the calculated effects t hat a re s ummarized i n T able 4 i n c olumn ' Co'. V ariables were recorded during 1 hour prior to a VAS fatigue rating (1h), 3 hours prior (3h), 6 hours prior (6h), since participants were awake (aw) and while they were asleep before a VAS fatigue rating (as).

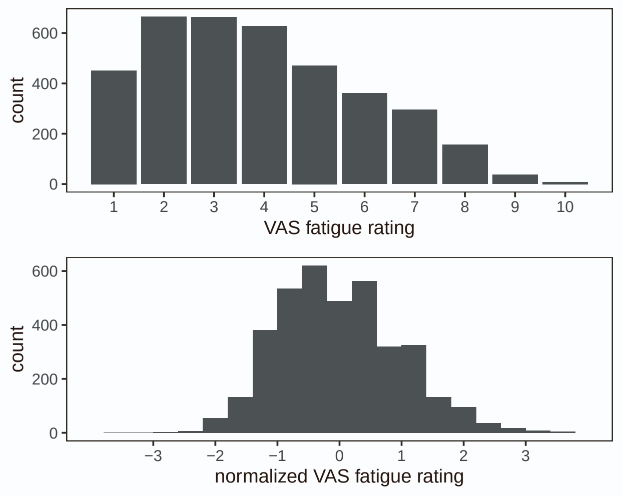

Figure S2: **Histogram of VAS fatigue rating as reported (top) and normalized per participant (bottom), related to Figure 1 and Section 5 (Method details).**
The continuous normalized VAS fatigue ratings are binned.

HRV metric			MS I	MS II	p
HR	asleep	min	47.1 (4.2)	48.1 (5.1)	0.383
HR	awake	min	52.4 (4.3)	53.8 (6.3)	0.345
HR	asleep	mean	62 (5.4)	63.2 (7.1)	0.633
HR	awake	mean	75.2 (5.6)	76.8 (8.3)	0.363
HR	asleep	max	99.1 (6.6)	99.4 (9)	0.933
HR	awake	max	118.1 (9.2)	116.8 (12.3)	0.437
SDNN	asleep	min	29.3 (14.4)	25.1 (10.4)	0.334
SDNN	awake	min	36.7 (11.9)	30.9 (10)	**0.053**
SDNN	asleep	mean	68.9 (24.1)	60.4 (20.1)	0.211
SDNN	awake	mean	64.4 (19.5)	56.7 (17.8)	**0.099**
SDNN	asleep	max	146.8 (42)	130.8 (33.4)	0.211
SDNN	awake	max	113.1 (31.5)	105.4 (32.7)	0.344
SD1	asleep	min	24.2 (14.1)	18.2 (10.5)	**0.078**
SD1	awake	min	26.6 (10.4)	20.2 (7.7)	**0.005**
SD1	asleep	mean	45.8 (25.1)	37.6 (19.5)	0.264
SD1	awake	mean	43.9 (16)	35 (14.3)	**0.022**
SD1	asleep	max	75.6 (38.2)	66.2 (30.8)	0.363
SD1	awake	max	66.8 (23.6)	55.7 (21.2)	**0.060**
SD2	asleep	min	33.9 (15.7)	30.5 (11.4)	0.715
SD2	awake	min	45.9 (14.9)	39.1 (12.2)	**0.081**
SD2	asleep	mean	88.7 (28.1)	78.7 (24.4)	0.198
SD2	awake	mean	84.4 (25.4)	75.1 (23.2)	0.145
SD2	asleep	max	201.3 (56.5)	179.4 (45.2)	0.211
SD2	awake	max	154 (43.3)	144.6 (45)	0.344

Table S3: **Mean (and SD) of HRV metrics for MS patients averaged while participants were awake and asleep, related to Table 1.**
This table relates to the split of MS patients into two subgroups in Table 1: MS patients with a functional ANS (MS I) and MS patients with a dysfunctional ANS (MS II). P-values corresponding to Wilcoxon signed rank tests are marked bold if they are smaller than 0.1.
