# Supplementary material for: Meaningful digital biomarkers derived from wearable sensors to predict daily fatigue in multiple sclerosis patients and healthy controls
Source: iScience. 2024 Jan 18;27(2):108965. doi: 10.1016/j.isci.2024.108965 (PMC10867654; doi:10.1016/j.isci.2024.108965)
Supplement: Document S2. PHRT author consortium [file mmc2.pdf]

MMAI-MS author consortium

Christian Holz (1), Gunnar R¨atsch (1), Fernando Perez-Cruz (2), Crist´obal Esteban (1), Martina Baumann (1), Rita Kuznetsova (1), Neda Davoudi (1), Shkurta Gashi (1), Liliana Barrios (1), Max Moebus (1), Ekaterina Krymova (2), Luis Salamanca (2), Firat Ozdemir (2), Marc Hilty (3), Veronika Kana (3), Patrick Roth (3), Andreas Lutterotti (3), (1): ETH Zurich, Department of Computer Science, Universit¨atstrasse 6, 8092 Z¨urich, Switzerland, (2): Swiss Data Science Centre, Turnerstrasse 1, 8092 Z¨urich, Switzerland, (3): University Hospital Zurich, Neuroimmunology Department, Frauenklinikstrasse 26, 8091, Switzerland
